# Allosteric Activation of Trypanosomatid Deoxyhypusine Synthase by a Catalytically Dead Paralog*[Fn FN3]^♦^

**DOI:** 10.1074/jbc.M113.461137

**Published:** 2013-03-21

**Authors:** Suong Nguyen, Deuan C. Jones, Susan Wyllie, Alan H. Fairlamb, Margaret A. Phillips

**Affiliations:** From the ‡ Department of Pharmacology, University of Texas Southwestern Medical Center at Dallas, Dallas, Texas 75390-9041 and; the § Division of Biological Chemistry and Drug Discovery, College of Life Sciences, University of Dundee, Dundee DD1 5EH, Scotland, United Kingdom

**Keywords:** Parasite Metabolism, Polyamines, Protozoan, Trypanosoma brucei, Trypanosome, Deoxyhypusine, Deoxyhypusine Synthase, eIF5A, Spermidine

## Abstract

Polyamine biosynthesis is a key drug target in African trypanosomes. The “resurrection drug” eflornithine (difluoromethylornithine), which is used clinically to treat human African trypanosomiasis, inhibits the first step in polyamine (spermidine) biosynthesis, a highly regulated pathway in most eukaryotic cells. Previously, we showed that activity of a key trypanosomatid spermidine biosynthetic enzyme, *S*-adenosylmethionine decarboxylase, is regulated by heterodimer formation with a catalytically dead paralog (a prozyme). Here, we describe an expansion of this prozyme paradigm to the enzyme deoxyhypusine synthase, which is required for spermidine-dependent hypusine modification of a lysine residue in the essential translation factor eIF5A. *Trypanosoma brucei* encodes two deoxyhypusine synthase paralogs, one that is catalytically functional but grossly impaired, and the other is inactive. Co-expression in *Escherichia coli* results in heterotetramer formation with a 3000-fold increase in enzyme activity. This functional complex is also present in *T. brucei,* and conditional knock-out studies indicate that both *DHS* genes are essential for *in vitro* growth and infectivity in mice. The recurrent evolution of paralogous, catalytically dead enzyme-based activating mechanisms may be a consequence of the unusual gene expression in the parasites, which lack transcriptional regulation. Our results suggest that this mechanism may be more widely used by trypanosomatids to control enzyme activity and ultimately influence pathogenesis than currently appreciated.

## Teaser

**Background:** Deoxyhypusine synthase (DHS) catalyzes the spermidine-dependent modification of translation factor eIF5A.

**Results:** Trypanosomatid DHS activity is increased 3000-fold by heterotetramer formation with a catalytically dead paralog, and both gene products are essential for parasite growth.

**Conclusion:** Trypanosomatid DHS is a complex between catalytically impaired and inactive DHS subunits.

**Significance:** This activation mechanism uniquely evolved for two independent enzymes within the trypanosomatid polyamine pathway.

## Introduction

Trypanosomatid parasites cause several fatal vector-borne human diseases, including the following: human African trypanosomiasis (HAT),^3^ American trypanosomiasis (Chagas disease), and leishmaniasis (1). Together, these parasites infect more than 20 million people primarily in tropical and subtropical regions. In particular, *Trypanosoma brucei gambiense* and *T. brucei rhodesiense*, the causative agents of HAT, are endemic in 36 countries in sub-Saharan Africa and are responsible for a debilitating neurological disease that invariably leads to death if untreated.

**FIGURE 1.**
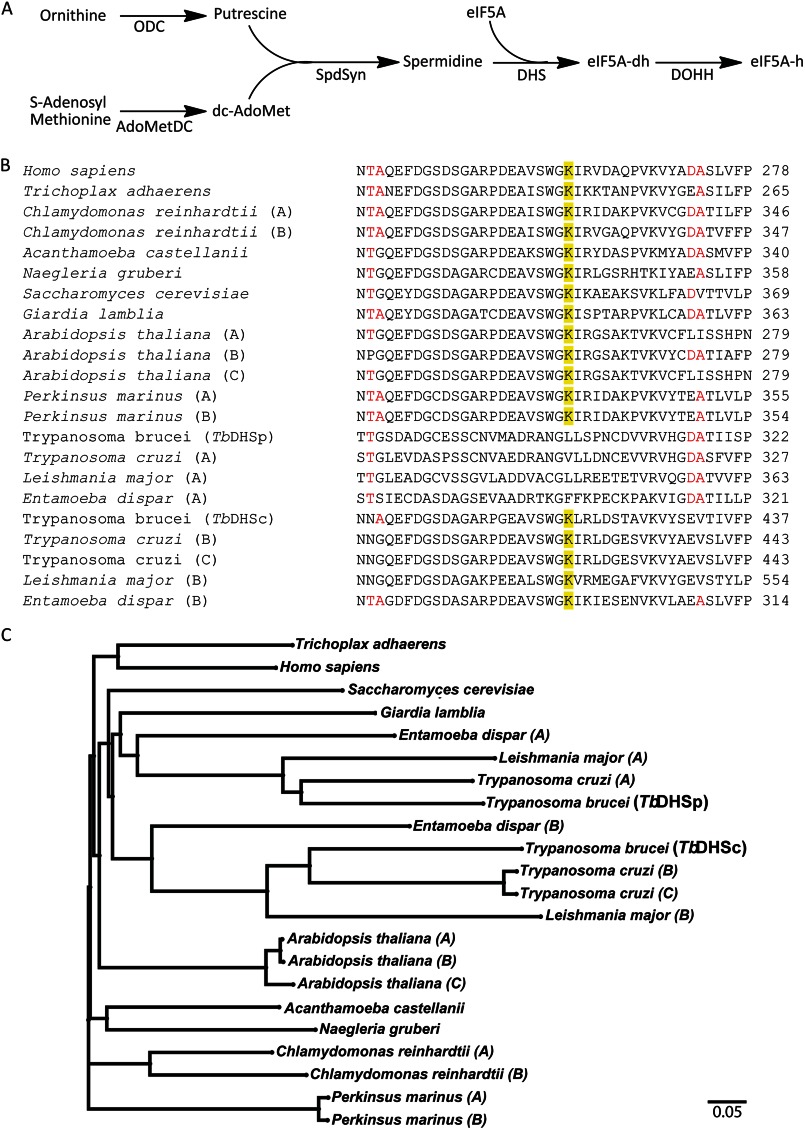
**Phylogenetic analysis of DHS genes in trypanosomatids.** *A,* spermidine and hypusine metabolic pathway in *T. brucei. B,* partial sequence alignment of DHS from select eukaryotes chosen to include a representative of each of the major eukaryotic lineages in the analysis: Opisthokonta (humans, *Trichoplax*, and *Saccharomyces*); Excavata (trypanosomatids, *Giardia*, and *Naegleria*); Amoebozoa (*Entamoeba* and *Acanthamoeba*); Archaeplastida (*Arabidopsis* and *Chlamydomonas*), and Alveolata (*Perkinsus*). Highlighted in *yellow* is the catalytic lysine residue. For organisms that contain more than one DHS homolog, duplicates are indicated using consecutive letters (*A–C,* etc.), except for those where function has been demonstrated in this paper (*e.g. T. brucei* DHSc and DHSp). Gene IDs are as follows: *Homo sapiens* (P49366); *Trichoplax adherens* (EDV28024.1); *Chlamydomonas reinhardtii* (*A,* EDP09680.1; *B,* EDP01029.1); *Acanthamoeba castellanii* (ELR12881.1); *Naegleria gruberi* (EFC43118.1); *Saccharomyces cerevisiae* (P38791); *Giardia lamblia* (EFO61259.1); *Arabidopsis thaliana* (*A,* AED90939.1; *B,* AAG53621.2; *C,* AED90940.1); *Perkinsus marinus* (*A,* EER15074.1; *B,* EER03596.1); *T. brucei* (*Tb*DHSp, Tb927.1.870; *Tb*DHSc, Tb927.10.2750); *T. cruzi* (*A,* Tc00.1047053511421.60; *B,* Tc00.1047053504119.29; *C,* Tc00.1047053506195.300); *Leishmania major* (*A,* LmjF.20.0250; *B,* LmjF.34.0330), and *Entamoeba dispar* (*A,* EDR24093.1; *B,* EDR21721.1). The full sequence alignment is shown in supplemental Fig. S1. *C,* Neighbor-Joining tree constructed with Mega5.

Eflornithine (difluoromethylornithine) is a suicide inhibitor of the polyamine biosynthetic enzyme ornithine decarboxylase (ODC) (Fig. 1*A*), which in combination with nifurtimox, is a front line treatment for HAT, demonstrating the importance of polyamine function for parasite growth (2). The cationic polyamines (putrescine and spermidine) are essential for growth of most eukaryotic cells and have been explored as potential targets for the treatment of both infectious disease and cancer (2, 3). Spermidine has been implicated in the regulation of translation and transcription, modulation of chromatin structure, and ion channel function (4, 5). In addition, in trypanosomatids spermidine is used in the synthesis of trypanothione (*N*^1^,*N*^8^-bis(glutathionyl)spermidine), required to maintain intracellular thiol-redox balance (6, 7).

Biosynthesis and metabolism of polyamines are tightly controlled; in mammalian cells regulation is orchestrated by a complex array of transcriptional, translational, and post-translational mechanisms (3, 4) that are generally lacking in trypanosomatids. Instead, these parasites have evolved a novel mechanism to control activity and expression of a key enzyme required for spermidine biosynthesis, *S*-adenosylmethionine decarboxylase (AdoMetDC) (2). Previously, we found that the functional trypanosomatid AdoMetDC was a heterodimer between a catalytically impaired subunit and a catalytically dead paralog, both of which were essential for cell growth (8, 9). We defined the term prozyme to describe activating subunits that arose via gene duplication of their partner enzyme. Heterodimer formation between AdoMetDC and the AdoMetDC prozyme led to a 1200-fold activation of AdoMetDC activity. Furthermore, the AdoMetDC prozyme protein levels appear to be translationally regulated, suggesting *T. brucei* modulates prozyme expression to control AdoMetDC activity and flux through the polyamine pathway (9).

**SCHEME 1.**
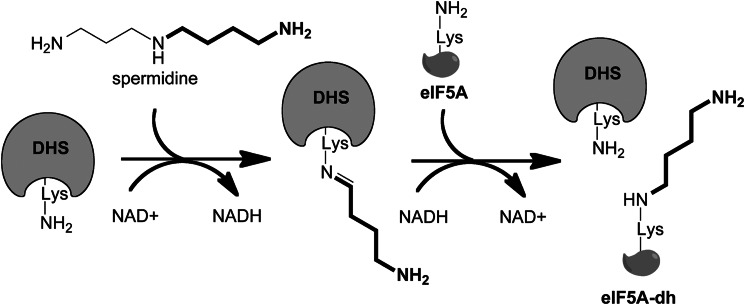
**Reaction mechanism of DHS.**

A specialized yet essential function of the polyamine spermidine in eukaryotic cells is to serve as a precursor for the hypusine modification of eukaryotic initiation factor 5A (eIF5A) (10). Hypusine-modified IF5A is present in both eukaryotes and archaea; although its functions are poorly understood, eIF5A is essential in yeast and mammalian cells (11). In bacteria, the eIF5A homolog elongation factor P, which is lysinylated instead of hypusinated, was shown to relieve ribosome stalling in the presence of polyproline tracks (12, 13). In yeast, eIF5A associates with translating ribosomes in a hypusine-dependent manner and is required for translation elongation (14, 15). Synthesis of hypusine requires two enzymatic reactions catalyzed by deoxyhypusine synthase (DHS) and deoxyhypusine hydroxylase. DHS catalyzes the modification of eIF5A to eIF5A-deoxyhypusine in a four-step NAD^+^-dependent reaction that proceeds through two imine intermediates (Fig. 1*A* and Scheme 1) (16). The reaction is highly specific and unique to eIF5A. The x-ray structure of human DHS (*Hs*DHS) shows the protein is a homotetramer formed from a dimer of dimers with each containing two active sites at the interface between monomers (17).

Genomes of kinetoplastids such as *T. brucei* and *Leishmania* species encode two homologs of *Hs*DHS, one of which appears to be catalytically dead. In *Leishmania donovani,* one of these homologs was shown to be essential and to encode a functional DHS, although it was significantly less active than the mammalian enzyme (18). The functional role of the second DHS homolog was not established. Here, we examine the roles of both homologs in *T. brucei* and demonstrate that both are required for optimal enzyme activity. Similar to AdoMetDC, we show that the two *T. brucei DHS* genes encode one catalytically active DHS subunit and one catalytically dead subunit that associate as a heterotetramer to form the active enzyme commensurate with a 3000-fold increase in catalytic activity. We also show that both genes are essential for parasite growth and infectivity *in vivo* and that the functional form of DHS in the parasite is the heterotetramer. These data demonstrate that the trypanosomatids have independently evolved an analogous strategy to activate two key enzymes involved in polyamine synthesis through oligomerization with a catalytically dead paralog. Trypanosomatids represent the only known species where this strategy is used to generate the catalytically active species of both DHS and AdoMetDC.

## MATERIALS AND METHODS

### 

#### 

##### Ethics Statement

Animal experiments were approved by the Ethical Review Committee at the University of Dundee and performed under the Animals (Scientific Procedures) Act of 1986 (UK Home Office Project License PPL 60/4039) in accordance with the European Communities Council Directive (86/609/EEC). To minimize animal suffering, mice with a terminal parasitemia (>10^8^ cells ml^−1^) were humanely killed.

##### Anti-DHS Antibody Production

Antibodies were raised in rabbits by Covance Inc., Denver, PA, against recombinant *Tb*DHSc and *Tb*DHSp purified from *Escherichia coli* (see below). Generation of rabbit polyclonal antibodies to *T. brucei* dihydroorotate dehydrogenase (*Tb*DHODH) was described previously (19).

##### Multiple Sequence Alignment

DHS sequences were obtained using NCBI BLASTP searches of the kinetoplastid protein database with *Hs*DHS (P49366) as the search query. Sequences were aligned with Clustal Omega (version 1.1.0). Phylogenetic trees were constructed with Mega5 software using the Neighbor-Joining algorithm with Kimura-2 parameters. DHS sequence accession numbers are listed in Fig. 1 and supplemental Fig. S1.

##### Cloning of TbDHSc, TbDHSp, and TbeIF5A

Genes (TriTrypDB accession numbers are as follows: *TbDHSp*, Tb927.1.870; *TbDHSc*, Tb927.10.2750; and *TbEIF5A*, Tb11.03.0410) were PCR-amplified from *T. brucei* single marker genomic DNA, cloned into pCR®8/GW/TOPO® (Invitrogen), and sequenced (Applied Biosystems Big Dye Terminator 3.1 chemistry and capillary instrumentation) to confirm that no mutations were introduced (see Table 1 for primers). No nucleotide polymorphisms were identified compared with the published genomic sequence of *T. brucei gambiense*. In addition, the 5′UTRs from both *TbDHSc* and *TbDHSp* genes were cloned by PCR from total RNA using the splice leader sequence as a forward primer and gene-specific reverse primers and sequenced in their entirety (Table 2).

**TABLE 1:**
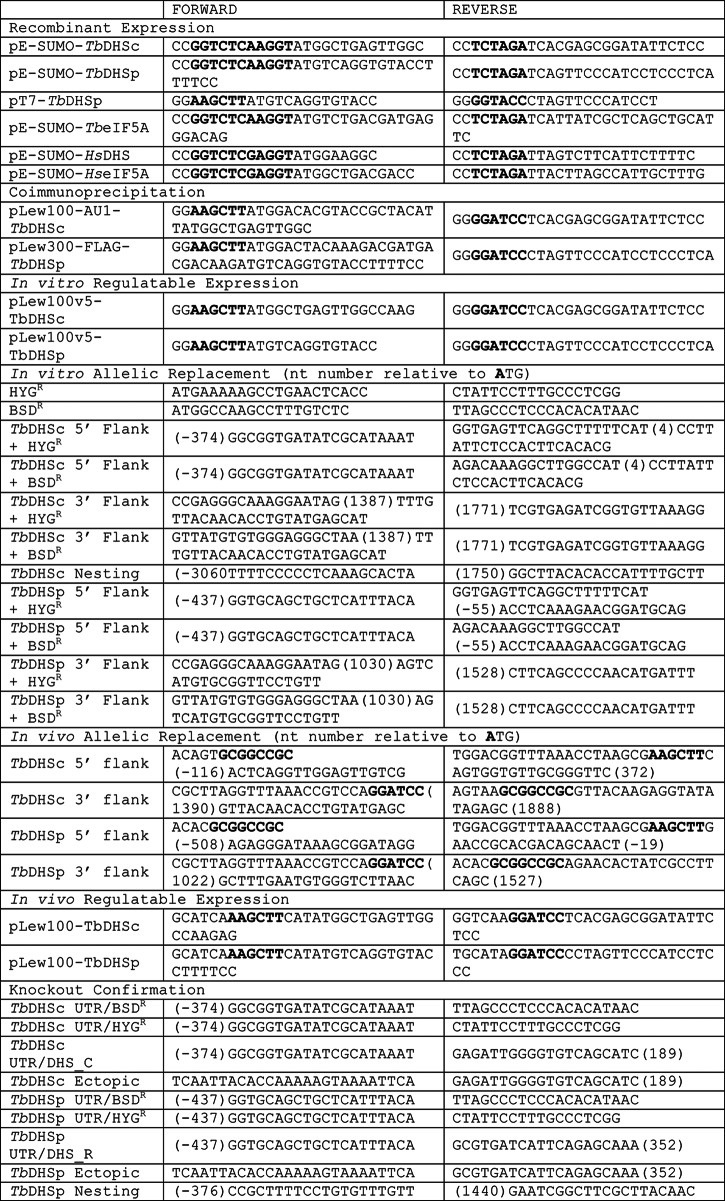
**Cloning primers** Restriction sites are shown in boldface type.

**TABLE 2:** **UTR sequence of *TbDHSc* and *TbDHSp*** 5′UTRs for *TbDHSc* (Tb927.10.2750) and *TbDHSp* (Tb927.01.870) were cloned by amplification from Tb427 cDNA using the splice leader sequence as a primer with a reverse primer in the ORF. Sequences of the cloned fragments are displayed. The spliced leader sequence is not shown.

**5′UTR for Tb927.1.870**
5′GCAGTGTCTACAACGCCATAAGGGGGGGGGGTGGTGTGCCGAGCTGTTTGTAGGGCGGCTGGAACGTT TGCAATAGGAGATCGGAAGCAGGAGGGTGATAGCAGTTATAGCCTTTACGGACCATCTTAAGTGAGAAATCCAACTTCAATT CTGCATCCGTTCTTTGAGGTACAAAAAACAAAAAGAGTGCGCACGGTGCCTTGGCAGTTGCTGTCGTGCGGTTC(ATG)
**5′UTR for Tb927.1.2750**
5′ACTACCGTTTCTTCCCTTCCGGGTCTTGCAACAGCTCGTGCTCTGAGGAAGTGCGTGTTTTGTCTTCA CATCACTTCGTTATTGGGCTTCTTTTCACCAACAACCAACACCACAAATCGTCACACCTCGTCACGCCGTCGCCGTTCTTGT AAATTTTTTTCTTCTGTCTAAGCCACTCAGGTTGGAGTTGTCGATTTTATCGCCAGGGAAAGCAGGATAAGTGGTAAGGGGA TAAAACTAAAAAGCCTCTTATTTGCAACTCCAGACCGCGTGTGAAGTGGAGAATAAGG(ATG)

##### In Vitro Growth and Transfection of T. brucei

Mammalian bloodstream forms (BSF) of *T. brucei* were cultured at 37 °C with 5% CO_2_ in HMI-11 media supplemented with 10% heat-inactivated tetracycline (Tet)-free fetal bovine serum (Atlanta Bio) as described (20). BSF single marker cells expressing T7 RNA polymerase and Tet repressor were used for genetic experiments (21). Transfection was performed with an Amaxa Nucleofector II as described (22) using NotI-linearized DNA (5 μg) followed by selection with the appropriate antibiotic. Antibiotic concentrations were as follows: 2 μg ml^−1^ G418 (Sigma), 2.5 μg ml^−1^ phleomycin (InvivoGen), 2 μg ml^−1^ blasticidin S (Sigma), 2 μg ml^−1^ hygromycin B (Sigma), and 1 μg ml^−1^ Tet (RPI). Cell growth was monitored with a hemocytometer; cell number was defined as cell density × culture volume × dilution factor. For GC7 EC_50_ determination, cell density was monitored using PrestoBlue (20 μl, Invitrogen) after 72 h of incubation with GC7 (0.01–1000 μm). Chicken serum was used for these studies to avoid polyamine oxidase-mediated toxicity of GC7. Fluorescence (560 nm excitation/590 nm emission) was measured using a Synergy^TM^ H1 Hybrid Multi-Mode Microplate Reader (BioTek), normalized to untreated cells, and data were fitted to Equation 1 using GraphPad Prism.

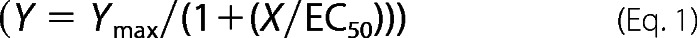


##### Generation of Tet-inducible TbDHSc and TbDHSp T. brucei Expression Constructs

Constructs were generated with and without N-terminal tags to allow expression of either native *Tb*DHSc and *Tb*DHSp, AU1-tagged *Tb*DHSc, or FLAG-tagged *Tb*DHSp. Forward PCR primers contained the DNA sequence of the desired tag as follows: AU1 tag (amino acids MDTYRYI) or FLAG tag (amino acid sequence MDYKDDDDK). Untagged genes and AU1-tagged *Tb*DHSc were then subcloned using HindIII and BamHI into pLew100v5 (phleomycin resistance (21)), whereas FLAG-tagged *Tb*DHSp was cloned into pLew300 (blasticidin resistance (9)). Both vectors allow integration of the plasmid into the rRNA locus and support Tet-inducible expression of the tagged gene in *T. brucei*.

##### Generation of T. brucei Gene Knock-out Constructs and Cell Lines

PCR fragments containing 5′- and 3′-flanking regions for the *TbDHSc* and *TbDHSp* genes on either side of the blasticidin or hygromycin resistance genes were generated by fusion PCR from *T. brucei* single marker genomic DNA. The 5′-flanking region of the *TbDHSc* (374 bp) or the *TbDHSp* genes (437 bp) were amplified with gene-specific primers (Table 1, flank primer sets). The reverse primer included an overhang complementing the blasticidin resistance gene (19 bases) or hygromycin resistance gene (21 bases). Similarly, the 3′-flanking regions of the *TbDHSc* (384 bp) or *TbDHSp* genes (499 bp) were amplified starting directly after the annotated stop codon, and the forward primer included an overhang to complement the resistance gene. The hygromycin resistance gene was amplified from the pLew90 vector (21) and the blasticidin resistance gene from the pLew300 vector (9). Amplified fragments were gel-purified and used in a second PCR with the *TbDHSc* or *TbDHSp* nesting primers (Table 1) and the amplified blasticidin or hygromycin resistance gene. PCR mixtures (50 μl) contained the following: 1× Phusion HF buffer, 200 μm dNTPs, 0.5 μm nesting primers, 20 ng of flanking fragment, 50 ng of resistance gene, and 1 unit of Phusion polymerase (New England Biolabs). PCR cycling conditions were as follows: 94 °C for 15 s, 65 °C for 30 s, and 72 °C for 2 min for 30 cycles. This reaction led to the joining of the *TbDHSc* or *TbDHSp* flanking regions in a cassette containing the resistance marker for replacement of the allele. The PCR product was gel-purified using the High Pure PCR product purification kit (Roche Applied Science) and transfected into *T. brucei* parasites directly.

For transfections, the first allele of the *TbDHSc* or *TbDHSp* gene was replaced with the hygromycin resistance gene, and clonal lines were obtained by limited dilution. The resultant single knock-out (SKO) cells were then transfected with the tagless *TbDHSc-* or *TbDHSp-*regulatable gene expression constructs and selected using phleomycin. Confirmation of ectopic expression upon Tet induction was obtained by quantitative PCR (qPCR) using a primer in the ectopic UTR and a primer within the gene (Table 1) and by Western blot using rabbit antiserum to recombinant *T. brucei* DHS. Expression levels from the Tet-induced ectopic copy were considerably higher than from the genomic DHS alleles. SKO cell lines that were confirmed to show good expression from the ectopic gene copy were then used to create conditional double knock-out (cDKO) cell lines by replacement of the second allele with the blasticidin resistance gene. Knock-outs were confirmed by PCR. Tet-regulatable cDKO cell lines were propagated in media containing Tet to maintain expression of *TbDHSc* or *TbDHSp* and with G418, hygromycin B, phleomycin, and blasticidin to maintain selection. To study the effects of DHS knockdown, *TbDHSc* and *TbDHSp* cDKO cells were washed three times with Tet-free media prior to plating in fresh media. Growth curves were analyzed for *n* = 3 biological replicates.

##### T. brucei Mouse Infection Model

**FIGURE 2.**
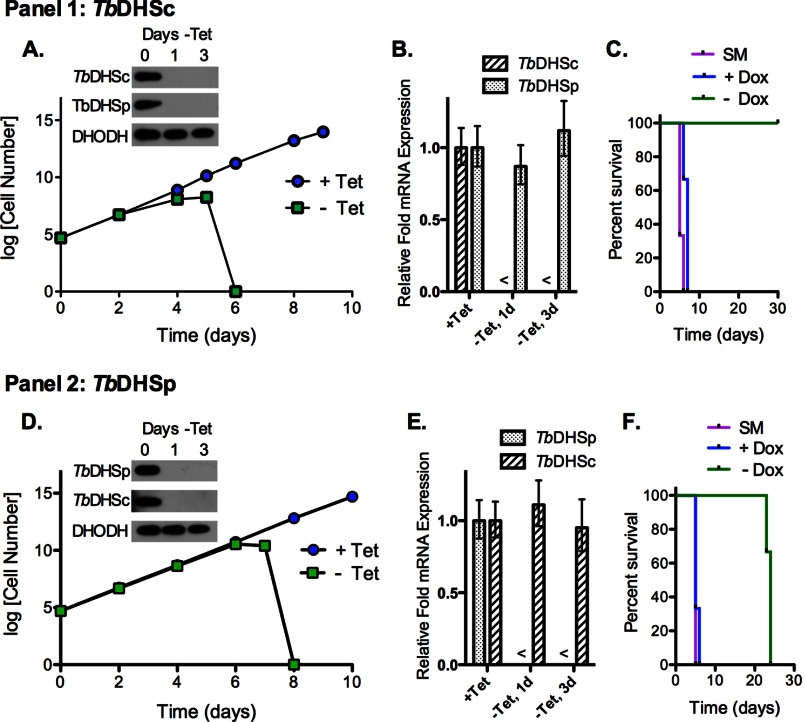
**Effects of DHS knockdown on *T. brucei* growth and survival.** *Panel 1,* effects of *TbDHSc* knockdown; *panel 2*, effects of *TbDHSp* knockdown. *A* and *D,* cell growth curve of log(cell number × dilution factor) over time. Data represent an average mean ± S.E. for multiple independent biological replicates. *A, TbDHSc* cDKO cells (*n* = 6); *D, TbDHSp* cDKO cells (*n* = 3); *blue circle*, + Tet (0.5 μg/ml); *green square*, −Tet. *Panel inset*, representative Western analysis performed with rabbit polyclonal antibodies to the indicated protein (30 μg of total protein); *Tb*DHODH was detected as a loading control. *B* and *E,* qPCR analysis of mRNA levels for *TbDHSc* cDKO cells (*B*) and *TbDHSp* cDKO cells (*E*). The symbol < indicates RNA levels were below the limit of detection. *Error bars* represent the mean ± S.D. for *n* = 3 replicates. *C* and *F,* Kaplan-Meier survival curves of infected mice (*n* = 3 per group). *C, TbDHSc* cDKO; *F, TbDHSp* cDKO cells; *SM* (single marker); *T. brucei* wild-type cells (*purple*), and cDKO-infected mice treated with (*blue*) or without (*green*) Dox.

A second independent set of cDKO clones was generated at University of Dundee and used for *in vivo* studies. Methods were essentially as described (23) using flanking primers and PCR (Table 1) to generate gene replacement constructs for *TbDHSc* and *TbDHSp* containing the hygromycin or puromycin resistance genes. These cell lines showed similar *in vitro* behavior to those characterized in Fig. 2, *A* and *C*. Wild-type and cDKO BSF *T. brucei* parasites were cultured in the absence of selectable drugs for 24 h with cDKO cells grown ±Tet. Cells were used to infect *n* = 3 mice per group (dosed ± doxycycline (Dox)) by a single intraperitoneal injection of 10^4^ parasites as described (24).

##### Recombinant Expression of DHS and eIF5A

*TbDHSc*, *TbDHSp*, and *TbEIF5A* genes were cloned into pE-SUMO Kan (Life Sensors) for expression as N-terminal His_6_-SUMO-tagged fusion proteins. PCR fragments generated with primers shown in Table 1 were digested with BsaI/XbaI and cloned directly into BsaI-linearized pE-SUMO. Untagged *TbDHSp* was also cloned into the HindIII-KpnI site of the pT7-FLAG^TM^-MAT-Tag®-2 vector (Sigma) where the C-terminal His tag was removed by insertion of a stop codon. *HsDHS* (P49366.1) and human *eIF5A* (*Hs*eIF5A) (P63241.2) sequences were codon-optimized for *E. coli*, synthesized by GenScript, and cloned into pE-SUMO Kan as above. Genes were expressed in T1 phage-resistant *E. coli* BL21(DE3) cells selected with kanamycin (50 μg ml^−1^) for single gene expression or kanamycin (50 μg ml^−1^) and ampicillin (100 μg ml^−1^) for co-expression of SUMO*-Tb*DHSc and *Tb*DHSp. Protein expression was induced at *A*_600_ of 0.5 with isopropyl β-d-1-thiogalactopyranoside (0.25 mm) for 16 h at 16 °C. Cells were harvested by centrifugation (1000 × *g* for 0.5 h), resuspended in Buffer A (50 mm Hepes, pH 8.0, 300 mm NaCl, 50 mm imidazole, 2 mm β-mercaptoethanol, 2 mm phenylmethylsulfonyl fluoride (PMSF)), and lysed by high pressure disruption (EmulsiFlex-C5, Avestin). Lysate was clarified (15,000 × *g* for 0.5 h), and protein was purified from the soluble fraction by Ni^2+^-affinity chromatography (HiTrap Chelating HP column, GE Healthcare) using a linear gradient from 50 to 320 mm imidazole in Buffer A for elution. SUMO tag was removed by treatment with Ulp1 (5 μg/ml final) (purified as described below) for 16 h at 4 °C. Sample was then diluted 20-fold in Buffer A, and the now tagless DHS was separated from the His_6_-SUMO by Ni^2+^-affinity chromatography. DHS-containing fractions (flow-through) were combined and dialyzed against DHS buffer (50 mm Tris-HCl, pH 7.5, 200 mm NaCl, 1 mm DTT). The *Tb*DHSc-*Tb*DHSp complex was further purified by gel filtration chromatography on a Superdex 200 Prep Grade (GE Healthcare) using DHS buffer. Purified protein concentrations were calculated using the following *A*_280_ extinction coefficients: *Tb*DHSc, 46.4 cm^−1^ mm^−1^; *Tb*DHSp, 25.9 cm^−1^ mm^−1^; *TbDHSc-Tb*DHSp, 72.3 cm^−1^ mm^−1^; *Tb*eIF5A, 4.1 cm^−1^ mm^−1^; *Hs*DHS, 39.9 cm^−1^ mm^−1^, and *Hs*eIF5A, 4.5 cm^−1^ mm^−1^ (computed using ProtParam, ExPASy, Swiss Institute of Bioinformatics).

##### Expression and Purification of Yeast SUMO Protease, Ulp1

The pET28b-Ulp1 expression construct was a gift from Dr. Kim Orth (University of Texas Southwestern). The protein was expressed with an N-terminal His_6_ tag in *E. coli* BL21 (DE3). Protein expression was induced as above except 1 mm isopropyl β-d-1-thiogalactopyranoside was used, and induction was for 2 h at 37 °C. Cells lysates were prepared as above and protein-purified by Ni^2+^-affinity chromatography as above except protein was eluted with Buffer A plus 250 mm imidazole in a single step.

##### DHS Enzyme Activity Assay

Activity was measured at 37 °C in 1-h reactions using a nitrocellulose filter binding assay to detect the incorporation of [^3^H]spermidine into eIF5A as described (25, 26). Reaction mixture (20 μl) contained recombinant DHS (4–40 nm *Hs*DHS, 10–40 μm *Tb*DHSc, or 5–20 nm *TbDHSc-Tb*DHSp), eIF5A (0.1–100 μm), and [^3^H]spermidine (7.5 μm hot or a hot-cold mixture ranging in concentration from 0.2 to 150 μm), NAD^+^ (0.5–1000 μm), DTT (1 mm), and glycine-NaOH buffer (0.2 m, pH 9.3). Initial rates of velocity data were fitted to the Michaelis-Menten equation using GraphPad Prism. The catalytic rate constant, *k*_cat_, was calculated based on active monomer concentration. For GC7 IC_50_ determination, velocity data were fitted to Equation 1 using GraphPad Prism. Data were collected in triplicate, and error represents the mean ± S.D.

##### Sedimentation Velocity

*Tb*DHSc-*Tb*DHSp complex (0.5 ml) was prepared at a range of concentrations (*A*_280_ 0.7, 0.2, and 0.05) in assay buffer (50 mm Hepes, 150 mm NaCl, pH 8.0) and then loaded into ultracentrifuge cells assembled with sapphire windows after overnight incubation at 4 °C. *A*_280_ and interference data were collected at 20 °C in an An-50 Ti rotor monitored continually for 16 h at 40,000 rpm in an Optima XL-I ultracentrifuge (Beckman-Coulter). Complete sedimentation was observed by 5 h. Interference data were analyzed using SEDFIT (27) to calculate sedimentation-coefficient distributions (*c*(*s*)) and estimate molecular weight. *c*(*s*) plots were generated in GUSSI.

##### Protein Quantitation

Protein concentration was quantitated using a protein assay (Bio-Rad) and a bovine serum albumin (BSA) standard curve, unless otherwise stated.

##### RNA and DNA Purification

RNA was isolated from *T. brucei* single marker BSF cells (2 × 10^8^ cells), washed in phosphate-buffered saline (PBS), pH 7.4, and then lysed using TRIzol reagent (Invitrogen) followed by purification with the RNeasy mini kit as recommended by the manufacturer (Qiagen). DNA was isolated from *T. brucei* single marker BSF cells (10^7^ cells) that were harvested and washed twice with PBS, pH 7.4, before being resuspended in DNA lysis buffer (1 m Tris-HCl, pH 8.0, 0.5 m EDTA, 5 m NaCl, 20% SDS, 0.1 mg of proteinase K) and incubated for 6 h at 55 °C. RNA was digested using RNase A at 37 °C for 30 min. An equivalent volume of chloroform was added, and the DNA was extracted in the aqueous layer, ethanol-precipitated, and resuspended in 50 mm Tris-HCl, pH 8.0.

##### Quantification of RNA Levels by qPCR

cDNA was synthesized from isolated RNA using the SuperScript®III first-strand synthesis system (Invitrogen). The reaction mixture (20 μl) contained the following: 2 μg of total RNA, 50 ng of random hexamers, 250 μm dNTPs, 1× RT buffer, 5 mm MgCl_2_, 10 μm DTT, 40 units of RNaseOUT^TM^, and 200 units of SuperScript^TM^III RT. RNA and primers were initially denatured at 65 °C for 5 min before the remaining components were added. The subsequent reaction conditions were as follows: annealing at 25 °C for 10 min, synthesis at 50 °C for 50 min, termination at 85 °C for 5 min, and removal of RNA with RNase H (2 units) at 37 °C for 20 min. The synthesized cDNA was used directly for qPCR without further purification. Relative gene abundance was quantified using the iQ^TM^ SYBR® Green Supermix and CFX 96-Real Time System by Bio-Rad. The reaction mix (20 μl) contained 100–150 ng of cDNA, 1× iQ^TM^ SYBR® Green Supermix, and 100 nm primers (Table 1). PCR cycling conditions included a one-time initial denaturation at 95 °C for 3 min followed by 40 cycles of the denaturation at 95 °C for 15 s and annealing/extension at 56 °C for 60 s. After completion of the cycles, melt curve analysis was done from 55 to 95 °C in 0.5 °C increments. Relative gene abundance was calculated by ΔΔ*Ct* using telomerase reverse transcriptase (TERT) as the reference gene (28).

##### Western Blot Analysis

Cells (typically 2 × 10^8^) were harvested by centrifugation (2000 × *g*, 10 min); pellets were washed twice with PBS, pH 7.4 (1 ml), resuspended in Tryp Lysis Buffer (50 mm Hepes, pH 8.0, 100 mm NaCl, 5 mm β-mercaptoethanol, 2 mm PMSF, 1 μg ml^−1^ leupeptin, 2 μg ml^−1^ antipain, 10 μg ml^−1^ benzamidine, 1 μg ml^−1^ pepstatin, 1 μg ml^−1^ chymostatin), and lysed with three freeze/thaw cycles. The lysate was clarified by centrifugation (13,000 × *g*, 10 min, 4 °C), and supernatant (30 μg of total protein) was separated by SDS-PAGE and transferred to a PVDF membrane (iBlot®, Invitrogen). The membrane was blocked with 5% milk in Tris-buffered saline (TBS) (20 mm Tris-HCl, pH 7.6, 137 mm NaCl) and incubated with primary antibody. Primary antibodies anti-*Tb*DHSc (rabbit polyclonal), anti-*Tb*DHSp (rabbit polyclonal), anti-FLAG/M2 (mouse monoclonal, Sigma), or anti-AU1 (mouse monoclonal, Covance) were used at a 1:1000 dilution, and rabbit anti-*Tb*DHODH was used at a 1:2500 dilution. Blots were washed with TBS + 0.1% Tween 20 and incubated with the appropriate secondary antibody at 1:10,000, goat anti-rabbit antibody or goat anti-mouse antibody conjugated to alkaline phosphatase (Sigma). Protein was detected using SuperSignal West Pico chemiluminescent substrate (Thermo Scientific). For a loading control, membranes were stripped and reprobed with antibody to *Tb*DHODH as described previously (19). Membranes were stripped with Restore Western blot stripping buffer (Thermo Scientific, Rockford, IL) for 20 min at RT and rinsed with TBS before blocking again with 5% milk in TBS.

##### Immunoprecipitation

BSF *T. brucei* cells (10^8^ cells) co-transfected with the AU1-*Tb*DHSc and FLAG-*Tb*DHSp expression plasmids were induced with Tet for 24 h before harvesting by centrifugation (2000 × *g*, 10 min). Cell pellet was washed twice with PBS, pH 7.4 (1 ml), resuspended in hypotonic buffer (10 mm Tris, pH 7.5, 2 mm PMSF, 1 μg ml^−1^ leupeptin, 2 μg ml^−1^ antipain, 10 μg ml^−1^ benzamidine, 1 μg ml^−1^ pepstatin, 1 μg ml^−1^ chymostatin), and incubated on ice for 1 h followed by three freeze/thaw cycles resulting in lysis and adjusted with salt buffer (10 mm Tris, pH 7.5, 400 mm NaCl) to 80 mm NaCl. Cell lysate was clarified by centrifugation (10,000 × *g*, 10 min, 4 °C). Total soluble protein (50 μg) was incubated alone or with either mouse monoclonal anti-AU1 antibody (Covance) or mouse monoclonal M2 anti-FLAG antibody (Sigma) (1:150 dilution for both antibodies) for 12 h at 4 °C. Dynabeads® protein A (50 μl, Invitrogen) was added, and the antibody-antigen complex was captured with a magnetic stand. The beads were washed three times with TBS, pH 7.6, and the antibody-antigen complex was eluted with 40 μl of citrate buffer, pH 3. Eluent was neutralized with 0.1 m NaOH (5 μl) before separation by SDS-PAGE and Western blot analysis as described above.

## RESULTS

### 

#### 

##### Phylogenetic Analysis of the DHS Genes in the Kinetoplastids

Two distinct clades of trypanosomatid DHS proteins were identified by BLASTP analysis of the translated trypanosomatid genome using *Hs*DHS as the search model (Fig. 1 and supplemental Fig. S1). Multiple sequence alignment and comparison of key residues show that one group consists of a protein that contains the catalytic Lys, shown to form the key imine intermediate with substrate (Scheme 1) (10, 17), while the other DHS group lacks the catalytic Lys despite containing many putative substrate-binding residues. The genes encoding these proteins are present on different chromosomes. We refer to the *T. brucei* gene products as *Tb*DHSc (Tb927.10.2750) reflecting the presence of the catalytic (c) Lys, and *Tb*DHSp (Tb927.1.870) where prozyme (p) designates an activating function. *Tb*DHSc exhibits 28% amino acid sequence identity with human *Hs*DHS but is 92 amino acids larger due to internal expansions. *Tb*DHSp shares 40% identity with *Hs*DHS but only 30% identity with *Tb*DHSc. *Trypanosoma cruzi* has two *DHSc* gene homologs (*Tc*DHS(B) and *Tc*DHS(C)) that are closely related and group together on the tree and one copy of the more diverged *DHSp* gene (*Tc*DHS(A)), whereas *Leishmania* species and *T. brucei* contain only a single copy of each gene. BLASTP analysis of eukaryotic DHS proteins showed that *Entamoeba* species also contain two significantly diverged paralogs of DHS, one with the catalytic Lys that groups with *Tb*DHSc and one without the Lys that groups with *Tb*DHSp. It is not clear if a single gene duplication event led to the generation of both the trypanosomatid *DHSp* and *Entamoeba DHSp* homologs or if they arose from independent events. All other eukaryotes appear to contain either only a single *DHS* gene or closely related gene duplicates that retain the catalytic Lys and are thus likely to be functionally equivalent and catalytically competent.

##### TbDHSc and TbDHSp Genes Are Essential for T. brucei Growth

We generated cDKO of the *TbDHSc* and *TbDHSp* genes in the *T. brucei* bloodstream form cells to determine whether one or both of the DHS genes were essential for cell growth. *T. brucei* is a diploid organism, so for both genes one endogenous locus was replaced with the hygromycin resistance antibiotic selection marker generating the SKO cell lines; a Tet-regulated copy of the respective DHS gene was inserted into the rRNA locus to serve as a rescue plasmid, and the second locus was then replaced with a blasticidin or puromycin resistance antibiotic selection marker generating the final cDKO cell lines. Independent cDKO lines were generated in each laboratory for each gene, and representative data are shown (Fig. 2). *TbDHSc* and *TbDHSp* cDKO lines were initially evaluated for growth defects *in vitro*. For *TbDHSc* cDKO cells, removal of Tet led to a >90% reduction in *TbDHSc* RNA and protein within 24 h, to a slowed growth by day 4, and to complete parasite clearing by day 6 (Fig. 2, *A* and *B*). Likewise, for the *TbDHSp* cDKO parasites, no detectable *TbDHSp* RNA or protein was observed 24 h after Tet withdrawal, and cell death occurred by day 8 (Fig. 2, *D* and *E*). Cultures were monitored by microscopy for an additional 4 days after cell death, and no live parasites were observed. These data demonstrate that both *Tb*DHSc and *Tb*DHSp are essential for survival of BSF *T. brucei in vitro*. Interestingly, in both cDKO cell lines, knock-out of one *DHS* gene (either *TbDHSc* or *TbDHSp*) led to the simultaneous loss of both *Tb*DHSc and *Tb*DHSp proteins (Fig. 2, *A* and *D*) despite the finding that the RNA as expected was only depleted for the gene targeted for knockdown (Fig. 2, *B* and *E*). These data suggested that *Tb*DHSc and *Tb*DHSp form a complex in the cell and that the individual proteins were not stable when the complex was disrupted.

##### DHS Is Essential for Infectivity of T. brucei in Mice

Mice were infected with *TbDHSc* and *TbDHSp* cDKO lines. One set of animals received Dox in their drinking water to maintain expression of the respective DHS proteins, and for the other set Tet was removed 24 h prior to inoculation, and mice were not administered Dox. Mice infected with *TbDHSc* or *TbDHSp* cDKO lines that received Dox in their water succumbed to parasitemia by day 6 after inoculation and showed an identical time course to mice infected with the control parental cell line (Fig. 2, *C* and *F*). In contrast, in the absence of Dox, mice infected with the cDKO of *TbDHSc* survived to the end of the experiment (day 30), at which time they remained parasite free and were assumed to be cured (Fig. 2*C*). Mice infected with cDKO of *TbDHSp* showed a prolonged survival time, but they eventually succumbed to parasitemia on day 24 after infection (Fig. 2*F*). The relapse of parasitemia in the *TbDHSp* cDKO infection suggests that a small number of parasites survived most likely through mutation in the Tet promoter, allowing re-expression of the *Tb*DHSp protein, as documented previously for other proteins with this system (21). These data demonstrate that *Tb*DHSc and *Tb*DHSp are essential to sustain an *in vivo* infection of *T. brucei* in mice.

##### TbDHSc and TbDHSp Form a Functional Complex

**FIGURE 3.**
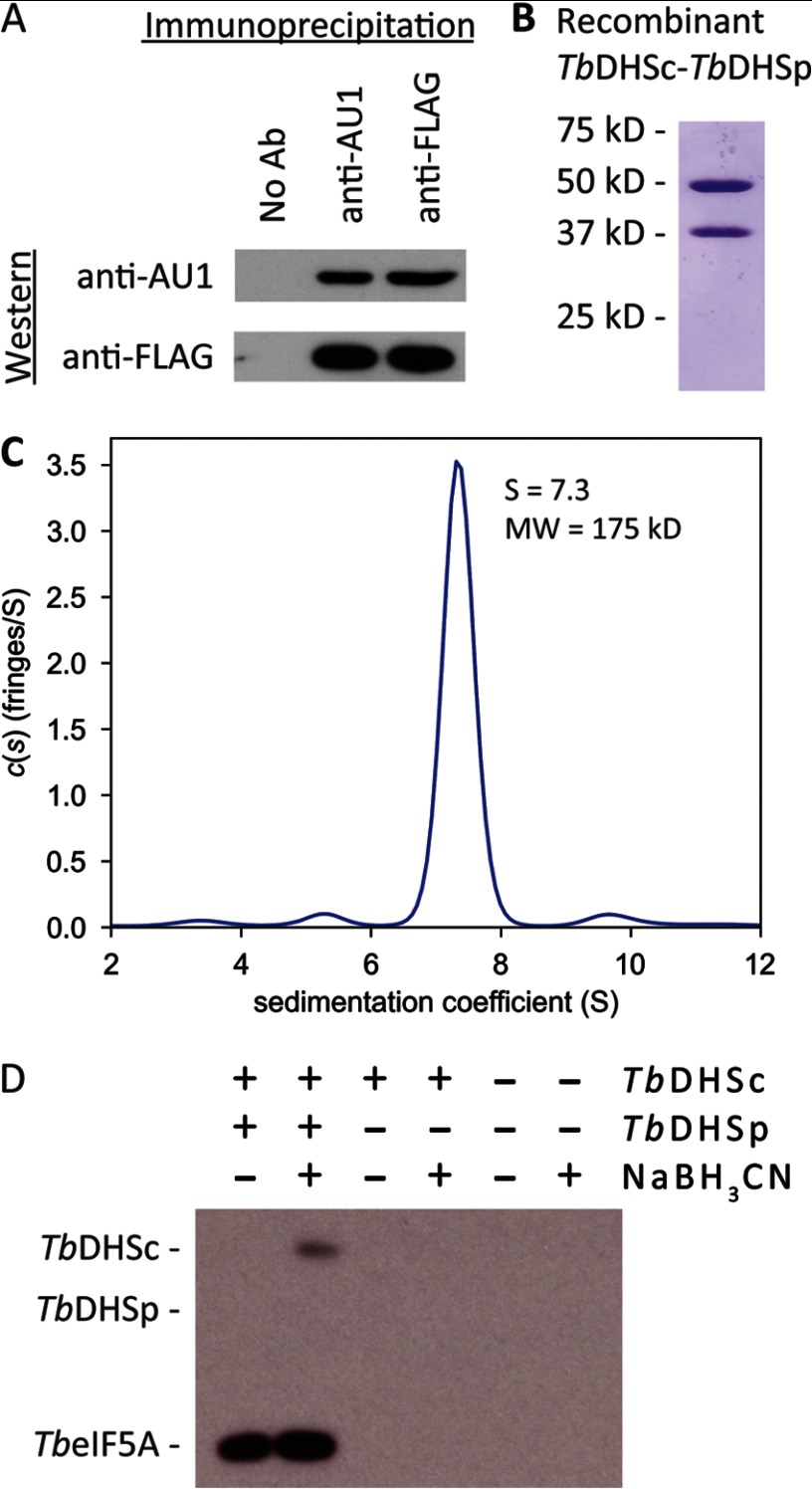
**Biochemical characterization of *T. brucei* DHS.** *A,* co-immunoprecipitation of AU1-*Tb*DHSc and FLAG-*Tb*DHSp from BSF *T. brucei*. Protein was immunoprecipitated with anti-AU1 or anti-FLAG antibody followed by Western blot analysis. *B,* SDS-PAGE analysis of *Tb*DHSc (50 kDa) and *Tb*DHSp (37 kDa) co-purified by Ni^2+^-affinity chromatography and gel filtration column chromatography. *C,* sedimentation velocity analysis of purified *Tb*DHSc-*Tb*DHSp complex. The observed *c*(*s*), signal population is shown as a function of *S. D,* NaBH_3_CN trapping of DHS reaction intermediates for *Tb*DHSc-*Tb*DHSp (0.1 μm) and *Tb*eIF5A (10 μm). Protein was separated by SDS-PAGE. [^3^H]Spermidine-labeled proteins were visualized by autoradiography.

To determine whether *Tb*DHSc and *Tb*DHSp form a complex, we generated a stable *T. brucei* BSF cell line that co-expressed N-terminally tagged AU1-*Tb*DHSc and FLAG-*Tb*DHSp. Immunoprecipitation of AU1-*Tb*DHSc from soluble *T. brucei* lysates using monoclonal antibody to AU1 was performed followed by Western blot analysis with anti-AU1 and anti-FLAG antibody. Both AU1-*Tb*DHSc and FLAG-*Tb*DHSp were found in the immunoprecipitate (Fig. 3*A*). Likewise, if a monoclonal antibody to FLAG was used for immunoprecipitation, both AU1-*Tb*DHSc and FLAG-*Tb*DHSp were detected (Fig. 3*A*). Thus, we can conclude that *Tb*DHSc and *Tb*DHSp form a protein complex in *T. brucei*.

To assess the activity of *Tb*DHSc and *Tb*DHSp, the open reading frames (ORFs) for these proteins and the substrate *T. brucei* eIF5A (*Tb*eIF5A) (Tb11.03.0410) were cloned into vectors for expression in *E. coli*, which is not capable of carrying out modification of eukaryotic eIF5A. *Hs*DHS and *Hs*eIF5A expression vectors were also generated to serve as controls. The proteins were expressed and purified as described under “Materials and Methods.” Unlike what was observed in *T. brucei*, both *Tb*DHSc and *Tb*DHSp could be expressed as stable proteins in *E. coli*. The ability of purified recombinant DHS to catalyze hypusine modification of eIF5A was measured with either *T. brucei* or human eIF5A as substrate using [^3^H]spermidine and a previously described filter binding assay (25, 26). The specific activity of purified *Tb*DHSc using *Tb*eIF5A as substrate was ∼10^3^-fold lower than the activity of *Hs*DHS on *Hs*eIF5A (Table 3), the latter being in agreement with previous reports (29, 30). The low observed activity of *Tb*DHSc was similar to that reported for the *Leishmania* enzyme (18). No activity was detectable for *Tb*DHSc with *Hs*eIF5A as the substrate. Recombinant *Tb*DHSp showed no activity within the limit of detection with either eIF5A substrate (Table 3).

**TABLE 3:** **Comparison of specific activity between DHS homotetramers and heterotetramers** Data were collected at fixed substrate concentrations (1 mm NAD^+^, 7.5 μm [^3^H]spermidine, and 10 μm eIF5A). Error represents the mean ± S.D. for six replicates.

	Specific activity	
	*s*^−*1*^	
Substrate	*Tb*eIF5A	*Hs*eIF5A
Enzyme		
*Hs*DHS	0.0029 ± 0.0001	0.016 ± 0.0003
*Tb*DHSc	1.8 × 10^−6^ ± 2 × 10^−8^	<10^−7^
*Tb*DHSp	<10^−7^	<10^−7^
*Tb*DHSc-*Tb*DHSp	0.0057 ± 0.0001	0.0035 ± 0.0001

To assess the activity of the *Tb*DHSc-*Tb*DHSp protein complex, tagless *Tb*DHSp was co-expressed with His_6_-SUMO-*Tb*DHSc in *E. coli*. Following purification by Ni^2+^-affinity chromatography, the SUMO tag was removed using Ulp1 protease and the tag-free protein complex further purified by size exclusion chromatography. *Tb*DHSc and *Tb*DHSp were present in approximately equimolar amounts in the peak fraction from this column confirming that the two paralogous gene products form a stable complex (Fig. 3*B*). Velocity sedimentation and analytical ultracentrifugation (Fig. 3*C*) revealed a single species of 175 kDa consistent with a 2:2 *Tb*DHSc-*Tb*DHSp heterotetramer. The specific activity of the heterotetramer was ∼3000-fold higher than for the *Tb*DHSc homotetramer, and it was functional on both *T. brucei* and human eIF5A substrates (Table 3). Substrate titrations were performed using the heterotetrameric *Tb*DHS (Table 4) and showed that the *k*_cat_ and the *K*_*m*_^app^ values for *Tb*eIF5A were similar to what has been reported for *Hs*DHS/*Hs*eIF5A, although the *K*_*m*_^app^ values for NAD^+^ and spermidine were ∼10-fold higher than reported for *Hs*DHS (30). These data demonstrate that the heterotetrameric *Tb*DHS complex is the functional enzyme in *T. brucei*.

**TABLE 4:** **Steady-state kinetic parameters for *T. brucei* heterotetrameric DHS** Variable concentrations of the substrate under determination were used with fixed concentrations (1 mm NAD^+^, 100 μm *Tb*eIF5A, and 100 μm spermidine) of the other substrates. Error represents the standard deviation for three independent experiments.

Substrate	*K*_*m*_^app^	*k*_cat_
	μ*m*	*s*^−*1*^
NAD^+^	82 ± 16	0.018 ± 0.001
*Tb*eIF5A	0.7 ± 0.1	0.018 ± 0.001
Spermidine	43 ± 5	0.015 ± 0.001

To further characterize *Tb*DHS activity, sodium cyanoborohydride was used to trap the imine reaction intermediates (Scheme 1). Reaction mixtures containing [^3^H]spermidine were treated with sodium cyanoborohydride, TCA-precipitated, separated by SDS-PAGE, and analyzed by autoradiography (Fig. 3*D*). In reactions containing *Tb*DHSc-*Tb*DHSp and *Tb*eIF5A, two bands were detected as follows: a strong band corresponding in size to *Tb*eIF5A, and a weaker band corresponding to *Tb*DHSc (Fig. 3*D*). These data show that as expected DHS is transiently labeled during the reaction and that the labeled substrate is transferred to *Tb*eIF5A resulting in deoxyhypusine modification (Fig. 1*A* and Scheme 1). No labeling of either *Tb*eIF5A or *Tb*DHSc was detected for reactions containing only *Tb*DHSc as the catalyst, again showing that on its own *Tb*DHSc is highly impaired in catalytic function and that only in complex with *Tb*DHSp is it fully functional.

GC7 is a structural analog of spermidine and a known inhibitor of *Hs*DHS (31). GC7 inhibited the activity of *Tb*DHSc-*Tb*DBHSp and the growth of BSF cells at similar concentrations (IC_50_ = 1.5 ± 0.15 μm and EC_50_ = 8.0 ± 1.5 μm, respectively). When AU1-*Tb*DHSc or FLAG-*Tb*DHSp was overexpressed in BSF cells independently, there was not a significant shift in the EC_50_ value for GC7 (EC_50_ = 5–6 μm). However, overexpression of AU1-*Tb*DHSc and FLAG-*Tb*DHSp together reduced sensitivity to GC7 (EC_50_ = 26 ± 3.0 μm), while *TbDHSc* and *TbDHSp* SKO lines were somewhat more sensitive (EC_50_ = 3.8 ± 0.4 and 5.5 ± 0.84, respectively). These data suggest that the mechanism of action of cell killing by GC7 is mediated by DHS inhibition, providing further evidence that *Tb*DHSc-*Tb*DHSp is the functional DHS species in *T. brucei*.

## DISCUSSION

Regulation and control of gene expression and modulation of enzyme activity are critical aspects of cellular function. Although diverse mechanisms for regulating enzyme activities are well known, we report here a new paradigm for potential enzyme regulation in the trypanosomatids based on activators that are catalytically dead enzyme paralogs termed prozymes. Remarkably, these parasitic protozoa have independently evolved this mechanism in two different steps in the same essential biochemical pathway, the biosynthesis of spermidine and the subsequent hypusine modification of a critical lysine in the translation factor eIF5A. Gene duplication of both trypanosomatid AdoMetDC and DHS led to the evolution of an enzyme activation mechanism in which one paralog retained limited catalytic function and the other lost key catalytic residues, but retained the ability to oligomerize with the catalytic subunit to greatly enhance catalytic activity. A pivotal feature of this model is that observed activation by the prozyme component is dramatically large (1000–3000-fold) and is thus likely to result from cooperative structural changes.

The functional significance of prozyme activation of DHS has been clearly demonstrated by our studies. We show that both *Tb*DHSc and *Tb*DHSp are essential for the growth of mammalian blood form *T. brucei* and for infection of a mammalian host. Additionally, GC7, a known inhibitor of DHS, was found to have anti-trypanosomal activity. We demonstrate that the functional species of DHS in *T. brucei* is a heterotetrameric complex between *Tb*DHSc and *Tb*DHSp and that complex formation is required not only for full activity but also for stability of the proteins in the parasite. These data genetically and chemically validate *T. brucei* DHS as a potential drug target and demonstrate the importance of the functional heterotetrameric DHS complex.

Despite the similarities to the AdoMetDC example, significant differences in mechanism are also present. The two DHS subunits are not stable in *T. brucei* unless in complex with each other. In contrast AdoMetDC prozyme and AdoMetDC levels are independent, and one can exist stably in excess over the other, which is a significant factor in AdoMetDC regulation in *T. brucei* (8, 9). *T. brucei* up-regulates AdoMetDC prozyme protein levels in response to inhibition or knockdown of AdoMetDC. It remains to be determined if *T. brucei* also regulates levels of DHSp to control deoxyhypusine formation in the parasite. Finally, although the AdoMetDC prozyme mechanism appears novel to the trypanosomatids, BLASTP analysis identified potential DHSp homologs in *Entamoeba* species in addition to the trypanosomatids. Functional analysis is needed to determine whether the *Entamoeba* DHSp paralog is also required to activate DHSc in this genus.

Our discovery that *T. brucei* DHS is activated by a prozyme mechanism adds to the list of unusual and novel mechanisms that cells have evolved to regulate polyamine metabolism or modulate enzyme activity. Polyamine metabolism is tightly regulated in mammals, plants, and yeast, although interestingly, no regulation of DHS has been described (3–5). Regulation occurs through common mechanisms such as transcriptional control but also through novel pathway-specific mechanisms. The intracellular turnover rate of ODC is controlled by expression of a protein inhibitor termed antizyme that targets ODC for degradation by the 26 S proteasome. Antizyme expression is in turn initiated by translational frame-shifting of antizyme mRNA when spermidine levels are high (32), and it is further regulated by the antizyme inhibitor, which is itself an inactive paralog of ODC (33). AdoMetDC expression is controlled by a small ribosome-stalling upstream open reading frame (uORFs) that is also sensitive to spermidine levels (34). Trypanosomatids not only lack these mechanisms but unlike other eukaryotes are also unable to regulate RNA polymerase II transcription (35–37). The protein coding genes typically lack introns (38) and are transcribed as large polycistronic clusters, which undergo 5′-leader splicing of the pre-mRNA (39). Regulation instead occurs during mRNA processing, mRNA degradation, translation, protein processing, and protein turnover (36). Furthermore, as a consequence of the mRNA trans-splicing reaction, 5′-UTRs are short, and translational control by uORFs has not been observed. Thus, the driving force to evolve novel mechanisms to regulate the polyamine pathway in trypanosomatids may have been the paucity of other potential mechanisms. Given the large investment made by cells to control and regulate polyamine levels, together with the number of novel mechanisms that have been uncovered, it is clear that regulation of this pathway is a key cellular function.

Inactive paralogs have been identified in a wide variety of gene families in metazoan species, although they are most prevalent in the kinase, protease, sulfotransferase, and RAS-like protein families (40–43). Inactive paralogs are perfectly poised to play regulatory roles, retaining the ability to bind both ligands and regulatory molecules. It has been shown that when duplicate genes evolve complementary mutations, the ability of cells to maintain both duplicates is enhanced, allowing novel function to evolve (44), thus providing a platform for the evolution of a regulatory function. With the exception of pseudo-kinases, there is still limited functional data on the roles of inactive paralogs. Examples of regulation by both inhibitory and activating mechanisms have been described, although most of the examples involve inhibition or dominant negative effects. The sheer magnitude of the activation observed for *T. brucei* DHS and AdoMetDC is unprecedented, and the observation that this occurs at two points in the same metabolic pathway is new.

In conclusion, the ability to regulate enzyme activity with a catalytically dead paralog provides cells with another tool for post-transcriptional regulation. Trypanosomatids represent the only known species where this regulatory strategy is available to potentially control the activity of DHS and AdoMetDC. Evolution of the prozyme mechanism in the trypanosomatids may have been driven by the need to control polyamine synthesis and function in an organism that lacks transcriptional control of gene expression and the frame-shifting and uORF-based mechanisms employed by many other eukaryotes. The discovery of this novel enzyme activation mechanism first for AdoMetDC and now for DHS powerfully confirms the importance of polyamines in the parasite, first exemplified by the discovery of trypanothione (6). Our data suggest that the paradigm of enzyme activation by a catalytically dead paralog may be more widespread than currently known. Indeed, many additional examples of this mechanism for the regulation of enzyme function in eukaryotes are likely still undiscovered.

## Supplementary Material

Supplemental Data

## Footnotes

3 The abbreviations used are:

HAT: human African trypanosomiasis
AdoMetDC: *S*-adenosylmethionine decarboxylase
ODC: ornithine decarboxylase
DHS: deoxyhypusine synthase
*Tb*DHSc: *T. brucei* deoxyhypusine synthase (catalytic subunit)
*Tb*DHSp: *T. brucei* deoxyhypusine synthase prozyme (activating) subunit
*Hs*DHS: human DHS
eIF5A: eukaryotic initiation factor 5A prior to hypusine modification
eIF5A-h: eIF5A with hypusine modification
BSF: bloodstream form *T. brucei*
GC7: *N*^1^-guanyl-*N*^7^-diaminoheptane
Tet: tetracycline
Dox: doxycycline
SKO: single knockout cell line
cDKO: Tet-regulated conditional double knockout cell line
qPCR: quantitative PCR.
