# Supplementary material for: Allosteric Activation of Trypanosomatid Deoxyhypusine Synthase by a Catalytically Dead Paralog
Source: J Biol Chem. 2013 Mar 21;288(21):15256–67. doi: 10.1074/jbc.M113.461137 (PMC3663545; doi:10.1074/jbc.M113.461137)
Supplement: Supplemental Data [file supp_288_21_15256__index.html]

Allosteric activation of trypanosomatid deoxyhypusine synthase by a catalytically dead paralog — Allosteric Activation of Trypanosomatid Deoxyhypusine Synthase by a Catalytically Dead Paralog — Allosteric Activation of Deoxyhypusine Synthase — Supplemental Data 

# Allosteric Activation of Trypanosomatid Deoxyhypusine Synthase by a Catalytically Dead Paralog

## Supplemental Data

**Files in this Data Supplement:**

- Supplemental Figure 1 (.pdf, 168 KB) - Supplemental Figure 1. DHS sequence alignment
