# Supplementary material for: Allosteric Activation of Trypanosomatid Deoxyhypusine Synthase by a Catalytically Dead Paralog
Source: J Biol Chem. 2013 Mar 21;288(21):15256–67. doi: 10.1074/jbc.M113.461137 (PMC3663545; doi:10.1074/jbc.M113.461137)
Supplement: Supplemental Data [file supp_M113.461137_jbc.M113.461137-1.pdf]

SUPPLEMENT

Allosteric activation of trypanosomatid deoxyhypusine synthase by a catalytically dead paralog

Suong Nguyen[1], Deuan C. Jones[2], Susan Wyllie[2], Alan H. Fairlamb[2], and Margaret A. Phillips[1]*

[1]Department of Pharmacology, University of Texas Southwestern Medical Center at Dallas, 6001 Forest Park Rd, Dallas, Texas 75390-9041, United State of America

[2]Division of Biological Chemistry and Drug Discovery, College of Life Sciences, University of Dundee, Dundee, United Kingdom

*Author to whom all correspondence should be addressed. Email: margaret.phillips@UTSouthwestern.edu; Tel: (214) 645-6164

Supplemental Figure 1. Sequence alignment of Deoxyhypusine Synthase from representative eukaryotes. Full sequence alignment of DHS from *Homo sapiens* (P49366), *Trichoplax adhaerens* (EDV28024.1), *Chlamydomonas reinhardtii* (A: EDP09680.1, B: EDP01029.1), *Acanthamoeba castellanii* (ELR12881.1), *Naegleria gruberi* (EFC43118.1), *Saccharomyces cerevisiae* (P38791), *Giardia lamblia* (EFO61259.1), *Arabidopsis thaliana* (A: AED90939.1, B: AAG53621.2, C: AED90940.1), *Perkinsus marinus* (A: EER15074.1, B: EER03596.1), *Trypanosoma brucei* (*Tb*DHSp: Tb927.1.870, *Tb*DHSc: Tb927.10.2750), *Trypanosoma cruzi* (A: Tc00.1047053511421.60, B: Tc00.1047053504119.29, C: Tc00.1047053506195.300), *Leishmania major* (A: LmjF.20.0250, B: LmjF.34.0330), and *Entamoeba dispar* (A: EDR24093.1, B: EDR21721.1). In human DHS, K329 is the catalytic Lysine (highlighted in yellow) and H288 is a crucial proton acceptor/donor (highlighted in cyan). In red text are residues involved in NAD$^+$ binding. Only residues that are conserved with the human enzyme are highlighted. Overall conserved residues are highlighted in green.

```
Homo sapiens               ----------------------MEGSLEREAPAGALAAVLKHSSTLPPE--STQVRGYDF
Trichoplax adhaerens       --------------------------MDNSTPSIAKEAVLVTSEAMPSD--AEAVKGYDF
S. cerevisiae              -----------------------MSDINEKLPELLQDAVLKASVPIPDD--FVKVQGIDY
Giardia lamblia            -----------MLVCVFIFKLLFFKGSQSMDEIQHASNNVIRASDTSCIGEKLEIHGLDL
Entamoeba dispar A         ----------------------------MSITGEEFAKVTSKVLGESKEYKGEPCIGYDF
Leishmania major A         MLASAPAPRPAKKDSAASRRKSASKSTGAAVKDDSSARVSASGAAESPEQSCTQVHGVDF
Trypanosoma cruzi A        ---------------------------------------------------METVDALDY
T. brucei (TbDHSp)         ----------------------------------------MSGVP----FPSRVIGDLDY
Entamoeba dispar A         --------------------------------------------------MTTTIKGYDF
T. brucei (TbDHSc)         ------------------------------MAELAKSAVLVSSCTDDLLGDAKQVVVGPN
Trypanosoma cruzi B        ------------------------------MAELAQKAVLIQSSDTNFQFHALGTVSGPA
Trypanosoma cruzi C        ------------------------------MAELAQKAVLIQSSDTNFQFHALGTVNGPA
Leishmania major B         ------------------------------MANIAESAVLVSSASSAQAVAKLTQVQGPT
Arabidopsis thaliana A     -------------------------MEDDRVFSSVHSTVFKESESLEGK--CDKIEGYDF
Arabidopsis thaliana B     -------------------------MEDDRVFSSVHSTVFKESESLEGK--CDKIQGYDF
Arabidopsis thaliana C     -------------------------MEDDRVFSSVHSTVFKESESLEGK--CDKIEGYDF
Acanthamoeba castellanii   ------------------------MDAHG--------FVMVKAEPLPEG--TPIVQGYDF
Naegleria gruberi          ------------------------MSQNTTTGDIGQQAVFIKTDPYECGLLQKEVRGYDF
C. reinhardtii A           --------------------------MAGCVDIPASQAVLVPTETIPDT---AVVRGYDF
C. reinhardtii B           --------------------------MATTDNKQGREAVLCATDVVPST---PVVKGFDF
Perkinsus marinus A        --------------MGADKSEEEESVASGQIPEIADDAVFLSSETV-DT---PVIQGYDF
Perkinsus marinus B        --------------MGADKSEEE-SVASGQIPEIADDAVFLSSETV-DT---PVIQGYDF


Homo sapiens               NRG-------VNYRALLEAFGTTGFQATNFGRAVQQVNAMIEKKLEPLS-QDEDQHADLT
Trichoplax adhaerens       NNG-------INHHSLLQSFRQTGFQATNFGLAIQEVNRMLELKAKPVS-EKDKNQLTLD
S. cerevisiae              SKPEATN---MRATDLIEAMKTMGFQASSVGTACEIIDSMRSWRGKHID--ELDDHEKKG
Giardia lamblia            NKPENQN-----LDAILSNYARMGFSSTGFSKLCNEVNRMYSWRLSDDP--YDPNRSYPE
Entamoeba dispar A         DNGVDFN-------KLMEKMKYTGFQALNLG----LCIEQVNEMR---------------
Leishmania major A         QSLVHATQE-ETLRAVVSSLPTTGLQATQIGRARQLVQQILHHR----------------
Trypanosoma cruzi A        SELVALNQE-EALRRVLASYPRIGLQATELGRARRIVQRALYHK----------------
T. brucei (TbDHSp)         SNLLNIGQE-EAIRCVLNAYPNIGLEATNLGRARRIVQRALNDNG---------------
Entamoeba dispar A         DKG-------VNYEELVNSYVTTGIQSSNVGRAINIINKMLTWQPSEEE-------KKEY
T. brucei (TbDHSc)         QED------LHSAEAVLNRYSTVGFQASNLARAFSICEMMLTPQSPSPSLMPTEGDQ---
Trypanosoma cruzi B        GDQ------LQSIAASLEHYAALGFQASHFSQAVAICKRMLQPQPPSVAVKQLTGSNDAN
Trypanosoma cruzi C        GDQ------LQSIAASLEQYAALGFQASHFSQAVAICKRMLQPQPPSAAVKQLTGSNDAN
Leishmania major B         SG-------FDKAQHIIGSYSTMGFQATNYGLARSIAQRMIRKQPPSKVYQIKDGKYVLV
Arabidopsis thaliana A     NQG-------VDYPKLMRSMLTTGFQASNLGEAIDVVNQMLDWRLADETTVAEDCSEEEK
Arabidopsis thaliana B     NQG-------VDYPKLMRSMLTTGFQASNLGEAIDVVNQMLDWRLADETTVAEDCSEEEK
Arabidopsis thaliana C     NQG-------VDYPKLMRSMLTTGFQASNLGEAIDVVNQMLDWRLADETTVAEDCSEEEK
Acanthamoeba castellanii   N-------KGLDYEALMASLKTTGFQATSFGQAVDEVNRMLRWSLNDEP-VTEKDDEESR
Naegleria gruberi          NNDLKDLNKPIDYNALLESYYTTGFQAHNFGEAVNILNAMLRWRLSDEP-MMENEQEPYD
```

```
C. reinhardtii A          NKG-------CDINGLMESMLTTGFQATTFGQAIAEVNRMINWRLSDEP-VGPATDPDHV
C. reinhardtii B          ATSR------PDLDNVMASMLTTGFQATSLGQAVNEVNRMIDWRLSDEP-VTADTPAEEA
Perkinsus marinus A       NNG-------VDFNAMMDQMMYTGFQATNLGLAFKQIDAMLDWSLNDEP-VADDEDEEFR
Perkinsus marinus B       NNG-------VDFNAMMDQMMYTGFQATNLGLAFKQIDAMLDWSLNDEP-VADDEDEEFR


Homo sapiens              --------QS-RRPLTSCTIFLGYTSNLISSGIRETIRYLVQH-----------------
Trichoplax adhaerens      --------AAGSRPLSNCSIFLGYTSNLISSGVRESIRYVVEH-----------------
S. cerevisiae             --------CFDEEGYQKTTIFMGYTSNLISSGVRETLRYLVQH-----------------
Giardia lamblia           --------CPVARSKIRCKIFLGYTSNLVSSGLREYIRFLVQH-----------------
Entamoeba dispar A        --------------KSHAKIFLGMSSNIVSSGLREVIHYLVKN-----------------
Leishmania major A        --------------SPEDRVFLAYTSNMISCGLRDTFTYLARE-----------------
Trypanosoma cruzi A       --------------RAGDAVFLAYTSNLISSGLRDTFACLARD-----------------
T. brucei (TbDHSp)        --------------MDGNKVMLAYTSNLISSGLRDTFACLARE-----------------
Entamoeba dispar A        --------VEGDERLKRCTIYLGFTSEMMTSGLRDTFRYLVEH-----------------
T. brucei (TbDHSc)        --------ASESPVMVQPTLFVGVTANLFGTGCREAIRFLCTECVPLP----NGVEPATP
Trypanosoma cruzi B       -----GKDASLTQVLVQPTIFLGATANLFGTGCREAIRFLCKESVSLP----HGVLPAAM
Trypanosoma cruzi C       -----GKDASLTQVLVQPTIFLGATANLFGTGCREAIRFLCKESVSLP----HGVLPAAM
Leishmania major B        PPDVGEDGRTLQQEHVYPNLFMGVSANLMGTGCREAVRFLVQEGVAHRSPEASAAASADG
Arabidopsis thaliana A    --------NPSFRESVKCKIFLGFTSNLVSSGVRDTIRYLVQH-----------------
Arabidopsis thaliana B    --------NPSFRESVKCKIFLGFTSNLVSSGVRDTIRYLVQH-----------------
Arabidopsis thaliana C    --------NPSFRESVKCKIFLGFTSNLVSSGVRDTIRYLVQH-----------------
Acanthamoeba castellanii  --------DPEYRKGVKCTIFLGYTSNMISSGVREIIRYLCQH-----------------
Naegleria gruberi         --------DPEVRKNTKCKVFLGYTSNMVSCGVREVIRFLVQH-----------------
C. reinhardtii A          --------DPAFRANTRCMIFLGFTSNLTSAGVREHIRYLVQN-----------------
C. reinhardtii B          --------DPEFRANARCIIFLGYTSNFTSAGTREQLRWLAQN-----------------
Perkinsus marinus A       --------SEEARLDVRTKVWLSYTSNIISSGCRELIRYIAEH-----------------
Perkinsus marinus B       --------SEEARLNVRTKVWLSYTSNIISSGCRELIRYIAEH-----------------


Homo sapiens              --------------------------------NMVDVLVTTAGGVEEDLIKCLAPTY---
Trichoplax adhaerens      --------------------------------NLVDCIVTTAGGIEEDFIKCLADTY---
S. cerevisiae             --------------------------------KMVDAVVTSAGGVEEDLIKCLAPTY---
Giardia lamblia           --------------------------------SLVDVIVASAGGVEEDIIKCLAPTY---
Entamoeba dispar A        --------------------------------KFVDAIVVTAGGIEEDFIKTMHPTL---
Leishmania major A        --------------------------------RLVDCFISSAGGIEEDVIKCGGSTL---
Trypanosoma cruzi A       --------------------------------RLIDGFISTAGGIEEDAIKCLGKTL---
T. brucei (TbDHSp)        --------------------------------NRIGAVVTTAGGVEEDVIKCLGDTL---
Entamoeba dispar A        --------------------------------KCVDYIVTTAGAIETDIMKCFGNIN---
T. brucei (TbDHSc)        LDDMAGIS-------CDGTGALKPS--PCDSRALIHVLVVSGGAMEHDIRRACESYKLSR
Trypanosoma cruzi B       PDEMSMPS-------CDIDDETIPLNPPFYSNALIHALVVSGGAMEHDIRRACEPYRITN
Trypanosoma cruzi C       PDEMSMPS-------CDIDDETIPLNPPFYSNALIHALVVSGGAMEHDIRRACEPYRITN
Leishmania major B        TDDQLMFARLKREYVETYGGPPHPDEEVPRAHSFLCAIVVSGGGVEHDLRRACTAYTLHY
Arabidopsis thaliana A    --------------------------------HMVDVIVTTTGGVEEDLIKCLAPTF---
Arabidopsis thaliana B    --------------------------------HMVDVIVTTTGGVEEDLIKCLAPTF---
Arabidopsis thaliana C    --------------------------------HMVDVIVTTTGGVEEDLIKCLAPTF---
Acanthamoeba castellanii  --------------------------------KLVDVIVSSAGGIEEDFIKCFAPTYC--
Naegleria gruberi         --------------------------------KLVDAIVTTCGAIEEDIMKTMQPTY---
C. reinhardtii A          --------------------------------RMVDVLVTTAGGIEEDFIKCMGHTY---
C. reinhardtii B          --------------------------------RMVDVMVTTAGGIEEDFIKCMANTY---
Perkinsus marinus A       --------------------------------HMAQVFITTAGGIEEDFIKCLADFH---
Perkinsus marinus B       --------------------------------HMAQVFITTAGGIEEDFIKCLADFH---


Homo sapiens              -----LGEFSLRGK----------------------ELRENGINRIGNLLVPNENYCKFE
Trichoplax adhaerens      -----IGDFRLPGR----------------------QLRDKGINRIGNLLAPNDNYCKFE
S. cerevisiae             -----LGEFALKGK----------------------SLRDQGMNRIGNLLVPNDNYCKFE
Giardia lamblia           -----LGDWRADGA----------------------MLRKNSINRIGNLLVPNDNYCLFE
Entamoeba dispar A        -----LGDFYFKGK----------------------ELYPNGYNRIGNLILPNSNYCEFE
Leishmania major A        -----LGQFGLDGR----------------------ALRRRGINRIGNLLVPNDNYCWFE
Trypanosoma cruzi A       -----VGQFSLDGR----------------------ELRRCGVNRTGNLLVPNDNYCHFE
T. brucei (TbDHSp)        -----VGDFALNDH----------------------ALRNNGLNRVGNLLVPNDNYRNFE
Entamoeba dispar A        -----IGDFYMKPE----------------------EVQGE---RHGNMIIPKEIIEKTK
```

```
T. brucei (TbDHSc)          DGAEEEGEQFHHPV-----------ERDRSRS------KGTDCH-FGNVRYNSSGVASRN
Trypanosoma cruzi B         YGG-FDGTPSHQ-------------QRQESAT------AGEDVARFGNISYGGSGTGTTS
Trypanosoma cruzi C         YGS-FDGTPSHQ-------------QRQESAT------AGEDVARFGNISYGGSGTGPTS
Leishmania major B          YASEAQGHVSSTISSEATAPLEGLQQRAETPLGTGAAAGAAKPARFGNVEYPPQG-SPGS
Arabidopsis thaliana A      -----KGDFSLPGA----------------------YLRSKGLNRIGNLLVPNDNYCKFE
Arabidopsis thaliana B      -----KGDFSLPGA----------------------YLRSKGLNRIGNLLVPNDNYCKFE
Arabidopsis thaliana C      -----KGDFSLPGA----------------------YLRSKGLNRIGNLLVPNDNYCKFE
Acanthamoeba castellanii    -----VGDFSLDGC----------------------ALRLKGQNRIGNLIIPNENYVKFE
Naegleria gruberi           -----LGAFDLDGK----------------------MLRLNGINRIGNLLIANQNYCKFE
C. reinhardtii A            -----LGDFQLKGS----------------------ELRMKGLNRIGNMVVPNSNYCKFE
C. reinhardtii B            -----LGDFHLKGE----------------------ELRKQGLNRIGNMVIPNANYCKFE
Perkinsus marinus A         -----LGDFALDGK----------------------TLRRRGLNRTGNLIVPNDNYCKFE
Perkinsus marinus B         -----LGDFALDGK----------------------TLRRRGLNRTGNLIVPNDNYCKFE

Homo sapiens                DWLMPILDQMVMEQNT---------------------EGVKWTPSKMIARLGKEINNP--
Trichoplax adhaerens        TWIMPILDQLVEEQNT---------------------HNINWTPSKIIARLGKEINNC--
S. cerevisiae               EWIVPILDKMLEEQDEYVKKH-----GADCLEANQDVDSPIWTPSKMIDRFGKEINDE--
Giardia lamblia             DWIIPIFDECMEQQRK----------------------GYHWTPSRLIWKLGERINDE--
Entamoeba dispar A          DWMDPLLLECLKQQNE---------------------HGVHWTPSKLVHKMGESINNE--
Leishmania major A          DFFTPVLESVQEAQRASR-------------------WKTHTAPSEFIEAMGAAIAKNH-
Trypanosoma cruzi A         NFFMPVLKHLHELQRESR-------------------WETMTAPSEMIAAIGAALGCKH-
T. brucei (TbDHSp)          DFFVPLLRRLHEQQRDSR-------------------WTTKTTPSQIIAEIGAALESVR-
Entamoeba dispar A          QWLKEFILDIQECQDT----------------------SMPFTPSQLITMMGERLNDT--
T. brucei (TbDHSc)          -LFSCVMRCLVKRLAEAQRKEKANREAAPIPEAYYDVCSWAITPSTLWYMAGLWMADIFT
Trypanosoma cruzi B         SIFTSVMRRLVSRLQAAQKRRKDASTAKPIPAAHDDVCEWAFSPSTVWYMTGRWLPELFT
Trypanosoma cruzi C         SIFTSVMRRLVSRLKAAQKRRKDASTAKPIPAVHGDVCEWAFSPSTVWYMAGRWLPELFT
Leishmania major B          ALFDRLMRTFAQRLCARQARLRAAAMAKPIPDKYDDVCSWSVTPSEVWALCGLWLVDMLA
Arabidopsis thaliana A      DWIIPIFDEMLKEQKE---------------------ENVLWTPSKLLARLGKEINNE--
Arabidopsis thaliana B      DWIIPIFDEMLKEQKE---------------------ENVLWTPSKLLARLGKEINNE--
Arabidopsis thaliana C      DWIIPIFDEMLKEQKE---------------------ENVLWTPSKLLARLGKEINNE--
Acanthamoeba castellanii    EWILPVLDAMVLEQKE---------------------KGEIWSPSKMISRFGKEINNP--
Naegleria gruberi           DWITPVLDAMLEEQY----------------------KGKLWSPSLMIDRFGKELNNE--
C. reinhardtii A            DWIIPILDACLTEQNE---------------------QGVNWTPSKLIDRLGKEIGHE--
C. reinhardtii B            DWMMPILDDMLKEQNE---------------------QGVNWTPSKIIARLGKEINDP--
Perkinsus marinus A         EWIEPIIDKMHDEQEQ---------------------DGVIWTPSTMIHRFGKEINDP--
Perkinsus marinus B         EWIEPIIDKMHDEQEQ---------------------DGVIWTPSTMIHRFGKEINDP--

Homo sapiens                -------------------------------------------------------ESVYY
Trichoplax adhaerens        -------------------------------------------------------NSVYY
S. cerevisiae               -------------------------------------------------------SSVLY
Giardia lamblia             -------------------------------------------------------RSIAY
Entamoeba dispar A          -------------------------------------------------------SSIYY
Leishmania major A          --------------------------------------------------PDTCTSSLVY
Trypanosoma cruzi A         --------------------------------------------------PETCSDSLLY
T. brucei (TbDHSp)          --------------------------------------------------PNDCGSSLIY
Entamoeba dispar A          -------------------------------------------------------TSVIT
T. brucei (TbDHSc)          EALQE-----------TGEVTDEKVAS--------------------EEGLKRAKSTVLY
Trypanosoma cruzi B         EVLRE-----------RSGGNMEAVA---------------------EEAQRRAESTVLY
Trypanosoma cruzi C         EVLRE-----------RSGGNMEAVA---------------------DEAQRRAESTVLY
Leishmania major B          EALRAVQSCPSHLTSGSGVGTAESVTANGKGQEADRDAHIATSALYRAEALARARTTVVY
Arabidopsis thaliana A      -------------------------------------------------------SSYLY
Arabidopsis thaliana B      -------------------------------------------------------SSYLY
Arabidopsis thaliana C      -------------------------------------------------------SSYLY
Acanthamoeba castellanii    -------------------------------------------------------ESVYY
Naegleria gruberi           -------------------------------------------------------DSILY
C. reinhardtii A            -------------------------------------------------------DSIYY
C. reinhardtii B            -------------------------------------------------------SSIYY
Perkinsus marinus A         -------------------------------------------------------RSVYY
Perkinsus marinus B         -------------------------------------------------------RSVYY
```

```
Homo sapiens               WAQKNHIPVFSPALTDGSLGDMIFFHSYKN------------------------------
Trichoplax adhaerens       WAYKNNIPVFSPALTDGSIGDMIYFHSYRN------------------------------
S. cerevisiae              WAHKNKIPIFCPSLTDGSIGDMLFFHTFKAS-----------------------------
Giardia lamblia            WAYRNKIPIFCPAITDGSLGDMLFFHSYKN------------------------------
Entamoeba dispar A         WAAKNNIPVFSPAITDGSIGDMIFFFSYKN------------------------------
Leishmania major A         WCYRNGISVFSPAFTDGSMGDMIYFYNFSH------------------------------
Trypanosoma cruzi A        WCYRNNIPVFSPALTDGSIGDMIYFYNYSK------------------------------
T. brucei (TbDHSp)         WCYRNDIPVFSPAFTDGSMGDMIYFYNYSR------------------------------
Entamoeba dispar A         WAAKNNITIFCPALTDGLFGTCITELNEIN------------------------------
T. brucei (TbDHSc)         WAARNGVPIFSPSLTDGDIMEFILTA----------------------------------
Trypanosoma cruzi B        WASMNGVPIFSPSFSDGDIMKFILDT----------------------------------
Trypanosoma cruzi C        WASMNGVPIFSPSFSDGDIMKFILDT----------------------------------
Leishmania major B         WAAVQQVSLFSPSFVDGDITSYLLPTPVPAARPAHRKGGSVADESAANSKELKRRRKASS
Arabidopsis thaliana A     WAYKMNIPVFCPGLTDGSLGDMLYFHSFRT------------------------------
Arabidopsis thaliana B     WAYKMNIPVFCPGLTDGSLGDMLYFHSFRT------------------------------
Arabidopsis thaliana C     WAYKMNIPVFCPGLTDGSLGDMLYFHSFRT------------------------------
Acanthamoeba castellanii   WCWKNDIPVFCPGLTDGSIGDMIYFHSYQN------------------------------
Naegleria gruberi          WAHKNEIPIYCPSLTDGSIGDMLYFHSYSDK-----------------------------
C. reinhardtii A           WAHKNNIPVFCPAITDGSIGDMLFFHSYKS------------------------------
C. reinhardtii B           WAYKNNIPVFSPAITDGSIGDMIFFHGKKN------------------------------
Perkinsus marinus A        WCYKNNIPVFCPAITDGSIGDMIYFHSYKR------------------------------
Perkinsus marinus B        WCYKNNIPVFCPAITDGSIGDMIYFHSYKR------------------------------

Homo sapiens               ----------------PGLVLDIVEDLRLINTQAIFA----------------KCTGMII
Trichoplax adhaerens       ----------------PGLRIDIVEDIRRMNSQAVFA----------------LNTGMLI
S. cerevisiae              --------------P-KQLRVDIVGDIRKINSMSMAA----------------YRAGMII
Giardia lamblia            ----------------PGLIIDVVGDIRAMNMQAVNS----------------PKNGCII
Entamoeba dispar A         ----------------EGLVLDLVQDVIKIDEMAFNA----------------EKVGCLL
Leishmania major A         ----------------KGLVVDPLEDVVRLRKLAAKE---------------KGRNLAIV
Trypanosoma cruzi A        ----------------KGLVLDPIVDVVRLRELGCRNRRCDDSQGGNRSQNNNGRTTCIV
T. brucei (TbDHSp)         ----------------KGLVVDPVPDVRRLRQLGCKS-------------TNVGRITCIV
Entamoeba dispar A         ---------------PVRLVVDLVQDLRLVNSSTIHS----------------VETGVII
T. brucei (TbDHSc)         -----------GDTGVPLLQLDLVADIHRLNRLAMRS----------------RRTGMMI
Trypanosoma cruzi B        -----------EDLTTAKLKLDLVVDIYRLNKFAMRS----------------QRSGMII
Trypanosoma cruzi C        -----------EDLTTALLKLDLVVDIYRLNKFAMRS----------------QRSGMII
Leishmania major B         SSPTAATAVEDEPPVVERLQIDLVRDVYSINKLAMLS----------------KKTGMLI
Arabidopsis thaliana A     ----------------SGLIIDVVQDIRAMNGEAVHAN---------------PKKTGMII
Arabidopsis thaliana B     ----------------SGLIIDVVQDIRAMNGEAVHAN---------------PKKTGMII
Arabidopsis thaliana C     ----------------SGLIIDVVQDIRAMNGEAVHAN---------------PKKTGMII
Acanthamoeba castellanii   ----------------EGLIVDIAQDIRGINNKAVYA----------------KKSGMII
Naegleria gruberi          --------------KGDGLVCDIVSDIRRLNGQAVRA----------------KKTGMVI
C. reinhardtii A           ----------------PGLRVDVVEDIRRINDIAMRAT---------------PRKTGMII
C. reinhardtii B           ----------------PGLRVDIAEDVARMNDIVLSAG---------------PRKTAMLL
Perkinsus marinus A        ----------------PGFIIDIAADIRKVNDESVKA----------------RHTGVIV
Perkinsus marinus B        ----------------PGFIVDIAADIRKVNDESVKA----------------RHTGVIV

Homo sapiens               LGGGVVKHHIANANLMRNG-ADYAVYINTAQEFDGSDSGARPDEAVSWGKIRVDAQPVKV
Trichoplax adhaerens       LGGGLVKHHICNANLMRNG-ADFSVFVNTANEFDGSDSGARPDEAISWGKIKKTANPVKV
S. cerevisiae              LGGGLIKHHIANACLMRNG-ADYAVYINTGQEYDGSDAGARPDEAVSWGKIKAEAKSVKL
Giardia lamblia            LGSGTIKHHILNANLFAGDGADFAVYINTAQEYDGSDAGATCDEAVSWGKISPTARPVKL
Entamoeba dispar A         VGAGIAKHHILNAMKRRGG-CDYCAMLSTSIECDASDAGSEVAADRTKGFFKPECKPAKV
Leishmania major A         LGGGLPKHHLLRNVS-----MDAVVMVTTGLEADGCVSSGVLADDVACGLLREETETVRV
Trypanosoma cruzi A        LGGGLPKHHLLQNVR-----ADTVVYVSTGLEVDASPSSCNVAEDRANGVLLDNCEVVRV
T. brucei (TbDHSp)         LGAGLPKHHLLRNVQ-----ADAVVYVTTGSDADGCESSCNVMADRANGLLSPNCDVVRV
Entamoeba dispar A         LGGGVMKHHIMNANLMRNG-ADFAVYINTAGDFDGSDASARPDEAVSWGKIKIESENVKV
T. brucei (TbDHSc)         LGGGVVKHHVCNANLMRNG-ADYAVFLNNAQEFDGSDAGARPGEAVSWGKLRLDSTAVKV
Trypanosoma cruzi B        LGGGVVKHHVCNANLMRNG-ADGAVFINNGQEFDGSDSGARPDEAVSWGKIRLDGESVKV
Trypanosoma cruzi C        LGGGVVKHHVCNANLMRNG-ADGAVFINNGQEFDGSDSGARPDEAVSWGKIRLDGESVKV
Leishmania major B         CGGGVVKHHVCNANLMRNG-ADFTIILNNGQEFDGSDAGAKPEEALSWGKVRMEGAFVKV
Arabidopsis thaliana A     LGGGLPKHHICNANMMRNG-ADYAVFINTGQEFDGSDSGARPDEAVSWGKIRGSAKTVKV
Arabidopsis thaliana B     LGGGLPKHHICNANMMRNG-ADYAVFINPGQEFDGSDSGARPDEAVSWGKIRGSAKTVKV
```

```
Arabidopsis thaliana C     LGGGLPKHHICNANMMRNG-ADYAVFINTGQEFDGSDSGARPDEAVSWGKIRGSAKTVKV
Acanthamoeba castellanii   LGGGLIKHHICNANLMRNG-ADYTVFINTGQEFDGSDSGARPDEAKSWGKIRYDASPVKM
Naegleria gruberi          LGGGVIKHHICNANLMRNG-ADFTVYINTGQEFDGSDAGARCDEAVSWGKIRLGSRHTKI
C. reinhardtii A           LGGGVPKHHICNANLMRNG-ADFAVYVNTAQEFDGSDSGARPDEAISWGKIRIDAKPVKV
C. reinhardtii B           LGGGVPKHHICNANLMRNG-ADFAVYLNTAQEFDGSDSGARPDEAISWGKIRVGAQPVKV
Perkinsus marinus A        IGGGVVKHHAMNANLMRNG-ADHVVYINTAQEFDGCDSGARPDEAVSWGKIRIDAKPVKV
Perkinsus marinus B        IGGGVVKHHAMNANLMRNG-ADHVVYINTAQEFDGCDSGARPDEAVSWGKIRIDAKPVKV


Homo sapiens               YADASLVFPLLVAETFAQKMDAFMHEKNED--------------------------
Trichoplax adhaerens       YGEASILFPLMVAETFAPVVEKMKTADHDNKRV-----------------------
S. cerevisiae              FADVTTVLPLIVAATFASGKPIKKVKN-----------------------------
Giardia lamblia            CADATLVFPLLLHETILKKYKEDPEYWDSKKGGDPHECYWTQMESEVRESKRS----
Entamoeba dispar A         IGDATILLPLIVASTFAKKEETTK--------------------------------
Leishmania major A         QGDATVVFPLMLI-AEKAATLEGAAA------------------------------
Trypanosoma cruzi A        HGDASFVFPLLLCKAETSADTHKDVAA-----------------------------
T. brucei (TbDHSp)         HGDATIISPLLLLRSSDGKEKVGVREDGN---------------------------
Entamoeba dispar A         LAEASLVFPLIVSKTFAVTKRFDGKI------------------------------
T. brucei (TbDHSc)         YSEVTIVFPLIVVHVFVAWVRMMR--SKGKENIRS---------------------
Trypanosoma cruzi B        YAEVSLVFPLLVAQVFLPFLRAARGVSLAEESEFL---------------------
Trypanosoma cruzi C        YAEVSLVFPLLVAQVFLPFVRAARGVSLAKESEFL---------------------
Leishmania major B         YGEVSTYLPLLVADVFVPAVRQRRATDDAQPRRRQSSRGARLPQDVSGHSHLCRGE-
Arabidopsis thaliana A     YCDATIAFPLLVAETFATKRDQTCESKT----------------------------
Arabidopsis thaliana B     YCDATIAFPLLVAETFATKRDQTCESKT----------------------------
Arabidopsis thaliana C     CFLISSHPNLYLTQWF----------------------------------------
Acanthamoeba castellanii   YADASMVFPLLVAETFVKHQLKKTKEQQQQEEGAKAQ-------------------
Naegleria gruberi          YAEASLIFPLLVAQTFVKYQ----REQEEKKLKEQQQ-------------------
C. reinhardtii A           CGDATILFPLLVSQTFVRHWTP--VEPLPEKKAEQE--------------------
C. reinhardtii B           YGDATVFFPLLLSQTFAKHFKPKGAGQAPQRRPESPNTPKVSGSGPTSAGI------
Perkinsus marinus A        YTEATLVLPLIIGKCFAPRVASGEWERTRGDGTRIVYNKSYTPSEHDKERRKLMAVN
Perkinsus marinus B        YTEATLVLPLIIGKCFAPRVASGEWERTRGDGTRIVYNKSYTPSEHDKERRKLMAVN
```
